# Mesoglea Extracellular Matrix Reorganization during Regenerative Process in *Anemonia viridis* (Forskål, 1775)

**DOI:** 10.3390/ijms22115971

**Published:** 2021-05-31

**Authors:** Maria Giovanna Parisi, Annalisa Grimaldi, Nicolò Baranzini, Claudia La Corte, Mariano Dara, Daniela Parrinello, Matteo Cammarata

**Affiliations:** 1 Marine Immunobiology Laboratory, Department of Earth and Sea Sciences, University of Palermo, 90123 Palermo, Italy; claudia.lacorte@unipa.it (C.L.C.); mariano.dara@unipa.it (M.D.); daniela.parrinello@unipa.it (D.P.); matteo.cammarata@unipa.it (M.C.); 2 Department of Biotechnology and Life Science, University of Insubria, Via Dunant 3, 21100 Varese, Italy; n.baranzini@uninsubria.it

**Keywords:** *Anemonia viridis*, regeneration, morphology, histology, enzymatic activity, collagen

## Abstract

Given the anatomical simplicity and the extraordinary ability to regenerate missing parts of the body, Cnidaria represent an excellent model for the study of the mechanisms regulating regenerative processes. They possess the mesoglea, an amorphous and practically acellular extracellular matrix (ECM) located between the epidermis and the gastrodermis of the body and tentacles and consists of the same molecules present in the ECM of vertebrates, such as collagen, laminin, fibronectin and proteoglycans. This feature makes cnidarians anthozoans valid models for understanding the ECM role during regenerative processes. Indeed, it is now clear that its role in animal tissues is not just tissue support, but instead plays a key role during wound healing and tissue regeneration. This study aims to explore regenerative events after tentacle amputation in the Mediterranean anemone *Anemonia viridis*, focusing in detail on the reorganization of the ECM mesoglea. In this context, both enzymatic, biometric and histological experiments reveal how this gelatinous connective layer plays a fundamental role in the correct restoration of the original structures by modifying its consistency and stiffness. Indeed, through the deposition of collagen I, it might act as a scaffold and as a guide for the reconstruction of missing tissues and parts, such as amputated tentacles.

## 1. Introduction

The ability to restore the correct tissue architecture after injuries requires a series of closely connected cellular and molecular events. Defects in the early steps of wound healing and the tissue remodeling program can interfere with regeneration [1], even in low invertebrates with a high regenerative capacity [2,3,4]. Therefore, understanding the mechanisms regulating tissue regeneration capacity can help shed light on factors related to the loss of vertebrate regenerative ability, which is reduced to only a few structures and tissues.

Although in the animal kingdom different mechanisms exist, in the last decades it has become clear that the extracellular matrix (ECM) plays a pivotal role during different physiological processes, including wound healing and tissue regeneration [5].

Since the Cnidaria, a sister group to Bilateria, possess high body regeneration capacities and can regrow missing body parts when dissected [4], is it is likely that knowledge in this group of organisms can drive the perspective on bilaterian regeneration.

Cnidarians are among the simplest living metazoans and evolved approximately 700 million years ago. They consist of two body layers derived from the endoderm (gastrodermis) and ectoderm (epidermis) of the embryo. Between these two cellular layers is an amorphous, jelly-like mesoglea connective layer. This generally contains no cellular elements other than some scattered amoebocytes and local genital cells.

The majority and most detailed cellular and molecular regeneration studies have been carried out using Hydra [6,7,8,9,10], and the colonial hydrozoan Hydractinia [11,12]. However, the regenerative potential has been described also in other medusozoa [13,14] and in anthozoan sea anemone *Nematostella vectensis* [1,15,16,17], in which a structure responsible for the repair and regeneration program and stem cell populations was identified [18].

Unlike the freshwater polyp Hydra, where regeneration is based on three distinct populations of multipotent stem cells, the I-cells, the ectodermal and endodermal epithelial cells [19,20,21,22,23], in anthozoans, another type of undifferentiated cell, called the amoebocyte, regulates this process [24,25,26,27]. These agranular hyaline cells are scattered in the mesoglea and can differentiate into fibroblast-like cells, characterized by extended pseudopodia and by the ability to control extracellular matrix production and collagen deposition [24].

The ultrastructure of the mesoglea in hexacoral anthozoan polyps shares some properties with that of various Hydra species, having a basal lamina composed of a lattice of fine filaments and containing collagen IV orthologs [28,29]. However, the role of this matrix in the regeneration of tissues, particularly in that of the tentacles, is not yet fully understood.

For this purpose, we used the Mediterranean *Anemonia viridis* as an in vivo model for the study of the matrix/cell interaction. 

*A. viridis* is a Mediterranean hexacoral anthozoan (from Atlantic Portugal up to the Irish coasts) belonging to the family Actinidae, that inhabits benthic rocky bottoms from 0 to 10 m deep. Like many anthozoans, it harbors zooxanthellae, photosynthetically active unicellular algae belonging to the genus Symbiodinium, within its gastrodermal cells. This association represents a trophic mutualistic endosymbiosis, in which zooxanthellae provide their cnidarian host with reduced organic carbon, resulting from their photosynthetic activity, while the host provides unicellular algae with inorganic carbon, inorganic nitrogen, and inorganic phosphate, as well as a refuge from herbivory [30,31]. The anatomy is distinguishable into three parts—the tentacles, the body column, in which the gastrovascular cavity is divided into several chambers by longitudinal partition called septa or mesenteries, and the base foot that binds to a solid surface.

The broader objective of the study was to analyze the mesoglea ECM, from a biochemical, biometric and morphological point of view, in unamputated and injured polyps after amputation of their oral tentacles. Specifically, we evaluated changes in mesoglea stiffness during regeneration, both in tentacles and the body’s mesenteries. The possible role of fibroblast-like cells, differentiated from hyaline amebocytes, in the remodeling of the ECM through the production of metalloproteases, the deposition and spatial organization of new collagen I fibrils in the mesoglea matrix were investigated. 

## 2. Results

### 2.1. Macroscopic Assessment of A. viridis Recovery 

The anemones survived the stress of injury. The tentacles hexamerously were arranged in several cycles moderately long (to 20–30 mm length), smooth, slender, tapering distally, inner ones longer than outer ones, contractile, dark-yellow to light-brown, sometimes with whitish or light pink tips.

**Figure 1:**
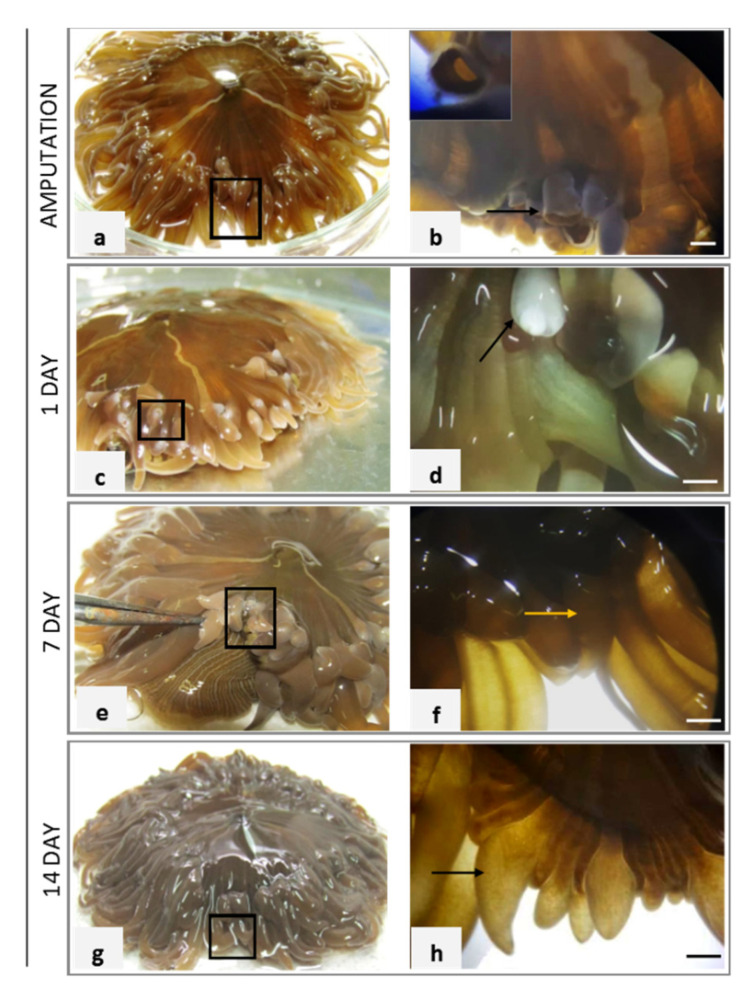
Morphological detection of the wound of *A. viridis* and regrowth of the amputated tentacles. Specimens injured in the black box area (**a**), wound area observed after 6 h with magnification of amputated tentacles indicated by the arrow (**b**). After 1 day post-amputation (dpa), the wound in the tentacles appear closed but avoid of pigment (**c**). Magnification of the closed terminal part of the tentacles with white pigmentation indicated by the arrow (**d**). At 7 dpa, the black boxed area includes the regenerated tentacles (**e**), and the small 2 mm long regrown tentacles showed a yellow–brown color like the global tentacular component (**f**). At 14 dpa, the tentacles continue to grow (**g**) in length and diameter with yellow–brown pigmentation (**h**). The arrow indicates a 4 mm long tentacle. Bar = 2 mm.

In Figure 1a, the black boxed area indicated where in the *A. viridis* adult specimen the tentacles were amputated. A lateral view of the amputated tentacle is shown in Figure 1b, in which the arrow indicates the magnification of the injury reported in inset.

After 1 day post-amputation (dpa), the injured terminal parts of tentacles appeared lighter in color than the rest of tentacles and the body (Figure 1c). The black box area indicated the non-pigmented area shown at a greater magnification in Figure 1d in which the terminal portion of the tentacle closed but without coloring is visible. The white pigmentation indicated by the arrow in the magnification is due to the absence the symbiont zooxanthellae in the wound closure area (Figure 1d). In the amputated specimens, a normal macroscopic tissue morphology was detected and pigmentation occurred within 7 days (Figure 1e). In Figure 1f, a visible tentacle bud of about 2 mm was indicated by the yellow arrow. The regenerative event appeared clear at macroscopic level after 14 dpa (Figure 1g) when a remarkable regrowth of about 3–4 mm was measured (Figure 1h).

### 2.2. Biochemical Response

**Figure 2:**
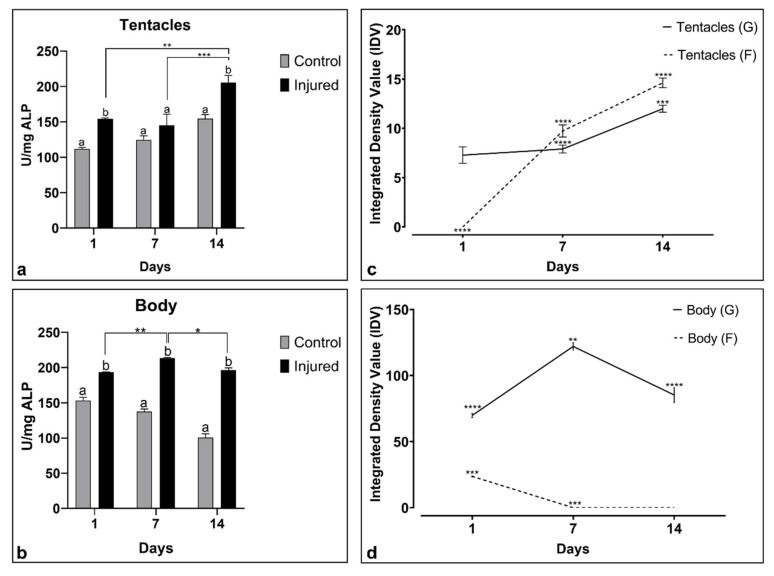
Alkaline phosphatase activity (ALP) and proteolytic activity in tentacles and in the body extracts of *A. viridis*. ALP in tentacles (**a**) and in the body (**b**) extracts of *A. viridis* of injured (amputated) and control samples. Kinetics of ALP was expressed as U*mg^−1^ of protein. Densitometric analysis of the proteolytic activity based on the integrated value of density (IDV) calculated on SDS-PAGE carried out with gelatin and fibrinogen as substrates using the tentacles (**c**) and the body tissue extracts of amputated specimens (**d**). Data were statistically analyzed using analysis of variance to determine differences between groups and showed mean ± standard error. The letters indicate statistically significant differences between control and its respective treated group. * Indicates differences between groups at each time. Significant differences were detected by Tukey post hoc test—* *p*  <  0.05, ** *p*  <  0.01, *** *p*  <  0.001, **** *p*  <  0.0001.

Alkaline phosphatase enzyme measured both in *A. viridis* tentacles and body extracts increased over time in amputated samples with respect to unamputated animals (Figure 2a,b). Particularly, in the tentacles (Figure 2a), the production of the enzyme constantly increased during the three observation step points. 

Multiple comparison analysis provided significant results for samples analyzed after 1 and 14 dpa with respect to control unamputated specimens.

On the other hand, in the body extracts (Figure 2b), the enzymatic values result was consistently and significantly higher than the control samples at all time step points. Significant results were detected for each treated group.

In both tissues, the two-way ANOVA analysis reported in Table 1 showed a *p*-value (<0.05) for the interaction of time (T) and injury (I) factors (TxI). In the body, both time (T) and injury (I) factors resulted in significance (*p*-value < 0.05) as well as their interaction (TxI).

The proteolytic activity was investigated in tentacles and body extracts using SDS-PAGE with gelatin and fibrinogen as substrates (Figure 2c,d). The gelatinolytic and fibrinolytic activity, assayed by the densitometric analysis and based on the calculated integrated value of density (IDV), grew over the time in the tentacles (Figure 2c). In the body, the fibrinolytic activity was present only at 1 day while the gelatinolytic activity was found from 1 to 14 dpa (Figure 2d). 

The two-way ANOVA results regarding proteolytic activity were reported in Table 2. A significant *p*-value (<0.05) in both body and tentacle tissue extracts was revealed for the variables Time (T), Substrate (S) and their interaction (TxS).

### 2.3. Biometry of Unamputated and Regenerating Tentacle Tissues

**Figure 3:**
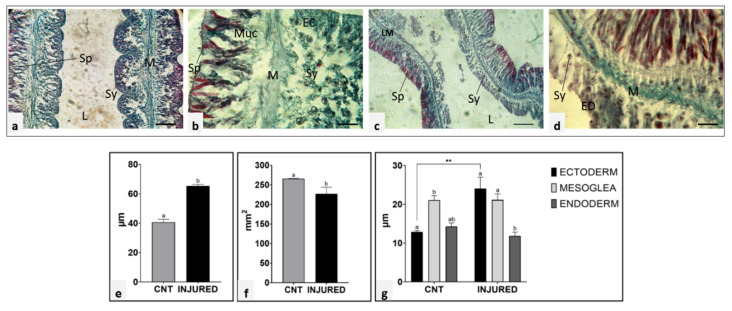
Gomori stain of tentacle transversal sections from uninjured specimens (**a**,**b**) and after 7 dpa (**c**,**d**). In light microscopy, Ectoderm (EC), Mesoglea (M), Endoderm (ED), Lumen (L), Spirocysts (Sp), Mucocytes (Muc), longitudinal muscle (LM), and *Symbiodinium* symbiont (Sy) were identified. The different dimensions of the tissue layers are evident in the magnifications of the sections of the control (**c**) and amputated (**d**) tentacles. Bars—50 (**a**,**c**) and 100 µm (**b**,**d**). Plots report that the biometric quantification of tentacular thickness increases (**e**) and the lumen decreases (**f**) in regenerating organisms post-amputation (injured) compared to the unamputated specimens (cnt). (**g**) shows the thickness of three body layers (ectoderm, mesoglea and endoderm). The Mesoglea and the Endoderm maintain constant size while the ectodermal layer increases in injured specimens with respect to the control (cnt). Analysis of variance was carried out. The letters (a,b) indicate statistically significant differences between control and its respective treated group. * Indicates differences between groups at each time. Significant differences were detected by Tukey post hoc test—** *p*  <  0.01.

In order to evaluate biometric parameters of regenerating tissues, longitudinal sections, stained with Gomori’s Trichrome, of control (unamputated) (Figure 3a,b) and 7 dpa tentacles (Figure 3c,d) were observed under a light microscope. Dimensional analysis of the different tissue layers in both control and post-injury specimens was carried out by image J software (Figure 3e–g). 

Of note, in the 7 dpa tentacle, both the ectoderm and the gastrodermis layers appeared thicker than in the unamputated tentacle, while the mesoglea size remained constant during all the regenerative phases. However, a pronounced variation in the mesoglea connective tissue density was appreciated. Indeed, after 7 dpa (Figure 3c,d) the connective tissue appeared more compact and darker green colored than that of the control tentacle. 

Comparing the control tentacles with the regeneration after amputation, a significant increase in tissue thickness (Figure 3e) and a reduction in lumen (Figure 3f), were observed. The thickness of the three tissue layers (ecto-, meso-, and endoderm) appeared significantly different between control (cnt) and amputated organisms (injured) (Figure 3g). Particularly, the ectoderm shows a statistically significant variation in the injured samples compared to the unamputated specimens.

### 2.4. Morphological and Ultrastructural Analyses of Regenerating Tentacle

In order to shed light on morphological modification occurring in the mesoglea connective tissue during tentacle regeneration, histological and ultrastructural analyses were performed on unamputated and regenerated tentacles. 

**Figure 4:**
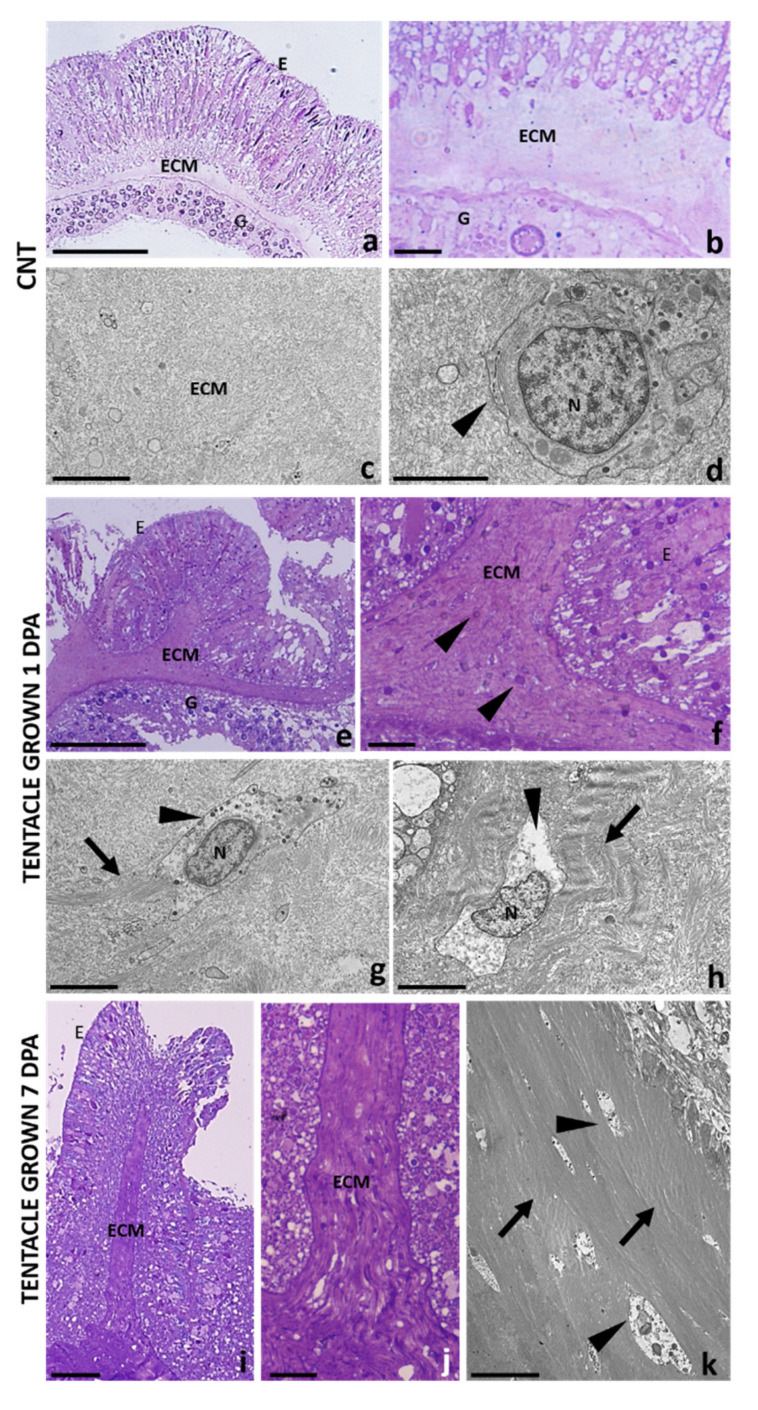
Light and electron transmission microscopy analyses of control and amputated organisms. In the control tentacle (**a**–**d**), mesoglea ECM, localized between the epidermis and gastrodermis, appears as a loose matrix. Ultrastructural TEM images (**c**,**d**) show that it is composed of a meshwork of thin filaments in which no fibrillar collagen and only few round shape amebocytes (arrowhead) are detectable. After one day from amputation (**e**–**h**), epithelial columnar cells of the regrowth tentacle are arranged in a circular way surrounding the inner mesoglea. Elongated amoebocytes (arrowheads) with irregular nuclei (**g**,**h**) and collagen fibrils (arrows) concentrated on their outer surface are visible. After 7 dpa (**i**–**k**), the regenerated tentacle increased in length, supported by a robust and extended collagenic scaffold, in which spindle-shaped fibroblast-like cells (arrowheads) have migrated. ECM, Mesoglea; E, Epidermis; G, Gastrodermis; N, nuclei. Scale bars—(**a**,**e**) 100 µm; (**i**) 50 µm; (**b**,**f**,**j**) 10 µm; (**c**,**k**) 5 µm; (**d**,**g**,**h**) 2 µm.

Control tentacle tissues revealed intact epithelia containing cells characteristic for each epithelium and separated by a loose mesoglea. Epidermis was formed by a single layer of densely packed columnar cells with the basal part mounted on the mesoglea. On the opposite side, the gastrodermis layer, with numerous endosymbiotic dinoflagellates zooxanthellae, was visible (Figure 4a). Mesoglea ECM appeared as an amorphous fibrous matrix (Figure 4b) in which no fibrillar collagen and only a few scattered rounded amebocytes containing a large nucleus were detectable (Figure 4c,d). 

The changes occurring in the ECM during the regenerative phase, both at the level of cellular and matrix reorganization, were evaluated in tentacles allowed to grow back for 1 and 7 days and subsequently cut from the base of the body. The epidermal layer of the tentacle regrown after one day from cutting, was formed by columnar cells arranged in a circular way and anchored with their basal side to the inner mesoglea (Figure 4e). An increased number of elongated amoebocytes with irregularly round nuclei appeared in the mesoglea protruding in the newly formed epidermal layer (Figure 4f–h). The darker violet staining (Figure 4e,f) indicated a connective tissue remodeling, also confirmed by ultrastructural electron transmission microscopy (TEM) analysis. Indeed, bundles of collagen fibrils were clearly detectable surrounding the elongated amebocytes, especially concentrated just to the outside of these cells (Figure 4g,h), strongly suggesting their fibroblastic nature.

After 7 days, the mesoglea infiltrating the newly formed epidermis increased in length together with the tentacle growth (Figure 4i). Bands of collagen fibrils were organized to form a robust scaffold in which aggregates of spindle-shaped fibroblast-like cells were observed (Figure 4j,k).

**Figure 5:**
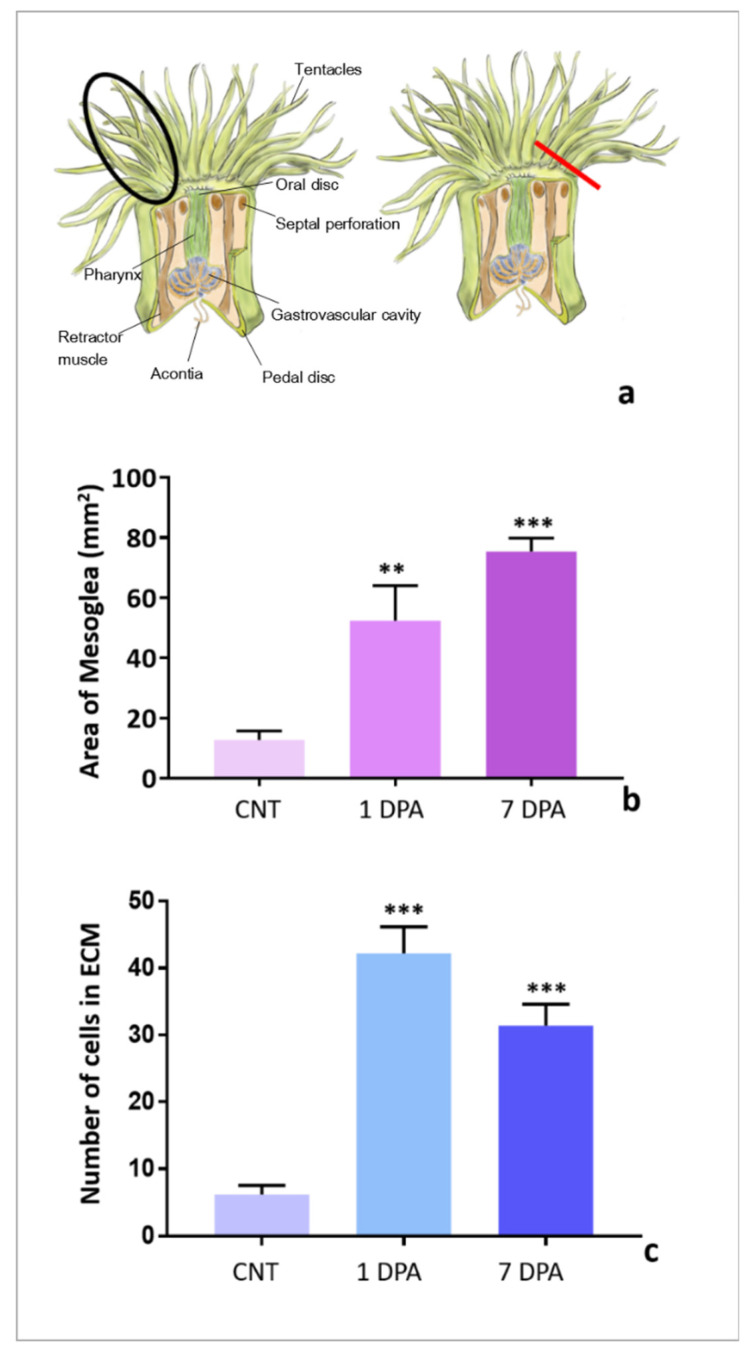
Comparison of ECM area and cell number between unamputated (black encircled) and amputated (red line) regenerating tentacles (**a**). The graphs show the quantification of the mesoglea area (**b**) and of elongated amoebocytes number (**c**) in noninjured (Cnt) and regenerating tentacles at 1 and 7 days post-amputation (dpa). Significant differences were detected by Fisher post hoc test—** *p*  <  0.01, and *** *p*  <  0.001.

The ECM area and the number of elongated amebocytes in both noninjured (Cnt) and amputated tentacles (Figure 5a) were quantified using ImageJ (see material and methods) and the graphs showed an increased ECM area after 1- and 7-days post-amputation (dpa) (Figure 5b). A greater number of elongated amebocytes was found 1 day after amputation.

These data suggested that these cells might be implicated in the ECM neo-synthesis and reorganization.

### 2.5. Mesoglea Matrix Reorganization and Collagen Neo-Synthesis

Based on the morphological observation, we were further interested in evaluating a possible interaction between tentacle regeneration and mesoglea expansion from the body’s mesenteries and whether the mesoglea ECM remodeling would be correlated to collagen neo-synthesis and fibroplasia events.

**Figure 6:**
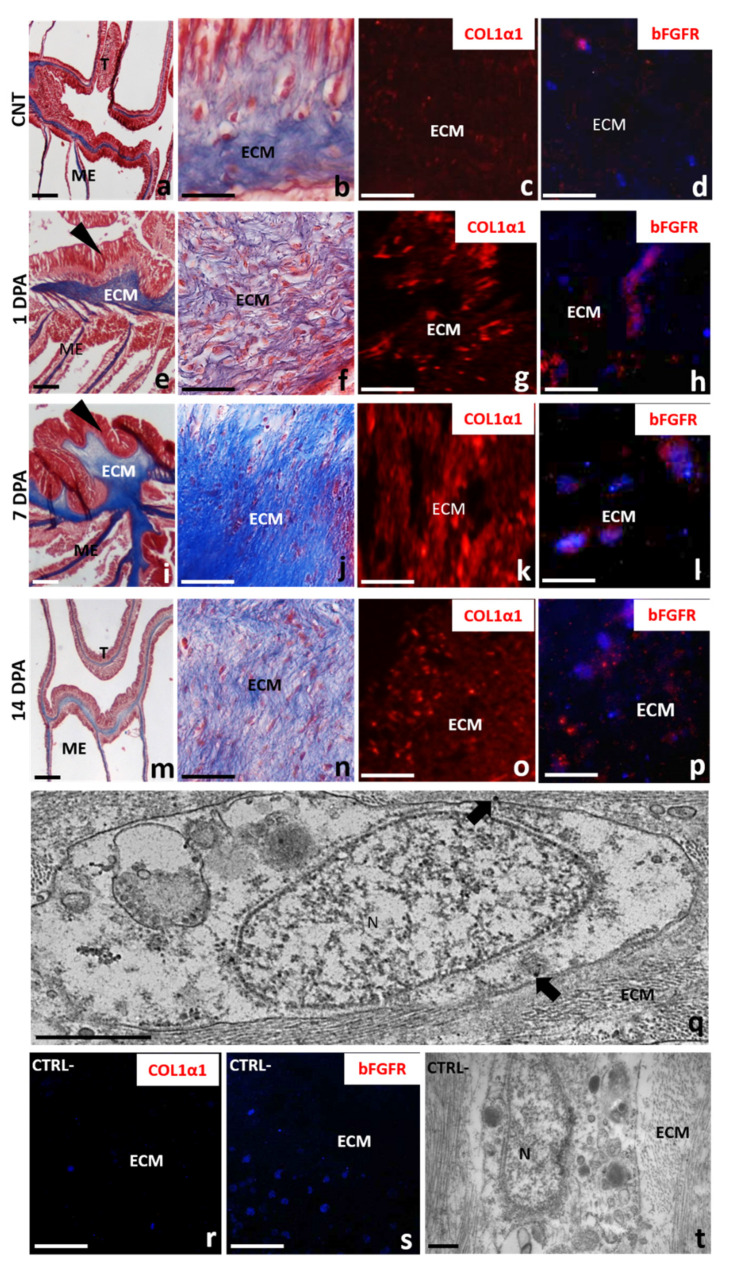
Masson’s trichrome and COL1α1/bFGFR immunolocalization assays. In control animals (**a**–**d**), Masson’s trichrome colorimetric assays highlighted the presence of a slightly colored ECM both in tentacles (T) and in the body wall (B) (**a**,**b**). A low immunofluorescent signal is detected for both anti-COL1α1 (red in **c**) bFGFR (red in (**d**)) primary antibodies, indicating the presence of few fibroblast-like cells involved in collagen production. After 1 dpa (**e**–**h**), an intensely blue-colored ECM (**e**,**f**), numerous dispersed COL1α1^+^ fibrils (**g**) and bFGFR^+^ fibroblast-like cells (**g**) are detectable in the tentacle bud (arrowhead) and in the mesenteries (ME). After 7 dpa (**i**–**l**), a compact scaffold formed by collagen fibrils in which aggregates of COL1α1^+^ (**k**) and bFGFR^+^ (**l**) spindle-shaped fibroblast-like cells are present in the ECM of both tentacle buds (arrow) and mesenteries. After 14 days (**m**–**p**), the ECM appears looser, less blue-staining (**m**,**n**) and low COL1α1 (**o**) and bFGFR (**p**) fluorescent signals are visible. (**q**) Immunogold localization at TEM showing the localization of bFGFR (arrows) on the fibroblast-like cell membrane. Nuclei are counterstained in blue by DAPI. No signals were detected in negative control experiments, in which primary antibodies are omitted (**r**–**t**). ECM, extracellular matrix of mesoglea; E, Epidermis; ME, mesenteries N-nuclei; T, tentacle. Scale bars—(**a**,**e**,**i**,**m**) 100 µm; (**b**,**f**,**j**,**n**,**r**,**s**) 20 µm; (**c**,**d**,**g**,**h**,**k**,**l**,**o**,**p**) 5 µm; (**q**,**t**) 500 nm.

To this aim, Masson’s trichrome staining with blue aniline, immunofluorescence and immunogold assays using anti-pro-collagen1α1 (COL1α1), to detect collagen I and bFGFR, a specific fibroblast marker in both vertebrates and invertebrates [32], was performed in the area of the body where the tentacles were cut and allowed to grow back for 1 and 7 days (Figure 6).

In both tentacles and mesenteries of healthy animals, the ECM had an amorphous and nonfibrotic appearance and was slightly colored by aniline blue (Figure 6a,b). Moreover, a low signal (Figure 5c) was detected for the antibody anti-COL1α1. 

By contrast, after 1 day from injury, a remarkable rearrangement of the mesoglea collagen-based connective tissue was evident. Indeed, the ECM, now more intensely colored in blue, was clearly organized in fibrils, both in the mesoglea of mesenteries adjacent to the regenerating area and in that infiltrating the tentacle bud, likely deriving from the mesentery itself (Figure 6e,f). Furthermore, COL1α1^+^ fibrils dispersed in the ECM were detected by immunofluorescence assay (Figure 6g). 

The fibrillar organization of the mesoglea matrix became even more evident especially in the sprawling bud regrown at 7 dpa, as also demonstrated by the significant increase of collagen I deposition (Figure 6i–k).

After 14 dpa (Figure 6m,n), the tentacle had almost completely regrown and the mesoglea matrix was looser both in the regenerated tentacle and in the mesenteries, appearing less intensely blue-colored and had a lower expression of COL1α1 (Figure 6o).

In order to better characterize the elongated and migrating amoebocytes found in the mesoglea connective tissue, immunofluorescence analysis using the anti-bFGFR antibody was performed. A basal level of bFGFR expression was found from a few resident cells located in the mesoglea of the control polyp (Figure 6d), whereas following tentacle amputation, an increased number of elongated bFGFR^+^ cells appeared in the ECM (Figure 6h,l,p) of both tentacles and mesenteries. The immunogold assay confirmed that the bFGFR was localized on the surface of hyaline amebocytes (Figure 6q), indicating the differentiation of these cells involved in new collagen production activity as fibroblast-like cells. In all the negative control experiments, no signals were detected (Figure 6r–t).

## 3. Discussion

In this study, we aimed to shed light on the structural and cellular mechanisms of the recovery of the mesoglea after injury, a key aspect of the regenerative process in cnidarians [33], using as a model the symbiotic anemone snakelocks *A. viridis*.

We first evaluated the regrowth of the oral tentacles after their amputation from the base of the body for 14 days, through a constant observation at a macroscopic level. At the same time, we quantified the activity of alkaline phosphatase and protease enzymes, typical biochemical markers associated with regenerative events. After 24 h, the closure of the wound is complete, and the injured part appears devoid of pigment probably due to the absence of zooxanthellae, lost from the tissues after injury. The sprawling regrowth was already noticeable after 7 days and new pigmented 5-mm-sized tentacles were visible after 14 days from injury. The return of pigmentation after 14 days is probably correlated to the repopulation of the tissues by the symbionts, which also seem to have an important role in the lengthening of the regenerating tentacles [34]. 

In vertebrates, one of the first events triggered during wound healing, inflammation and regeneration, is the alkaline phosphatase (ALP) activity, especially by fibroblasts localized in the connective tissue [35]. This enzyme is usually highly expressed also during tissue regeneration in cnidarian and in Hydra, and it has been described as one of the main enzymatic markers related to nematocyte and epithelial cell differentiation during tentacle regeneration [36].

Here, we showed that ALP in *A. viridis* increases significantly in tentacle extracts after amputation in all three time points considered, while the enzyme amount remains at a constant level in the body extract. 

Algae convert dissolved organic phosphorus (DOP) in the environment into dissolved inorganic phosphorus (DIP), which is used by animal organisms for their metabolic activities. The symbiotic zooxanthellae, abundant in the tentacles, play a crucial role in the absorption of phosphate dissolved in water [37,38,39,40]. The different amount of ALP present in the tentacles compared to the body may be explained with their more effective absorption and bioaccumulation capacity of phosphorus. 

Wound healing, occurring immediately after an injury and involving cell contraction and re-adhesion, is the phase that precedes the regeneration of the lost structures. During both of these phases, the immune response facilitates protection against microbes, maintaining homeostasis and preventing infections that might compromise fitness [41,42]. Alongside immune system activation, in response to injury, protease enzymes, normally minimally active in adult tissues are upregulated during regeneration and tissue remodeling [43].

Matrix metalloproteinases (MMPs) are a family of endopeptidases collectively able to degrade the components of the extracellular matrix. They play important roles in propeptides and collagen fibril formation and normal tissue remodeling, embryogenesis, angiogenesis, and wound healing [27,44].

In cnidarian, several typical components of vertebrate ECM, such as fibronectin, laminin and different types of collagen are involved in cell adhesion [45,46,47,48,49,50], and several matrix proteases with gelatinolytic and fibrinolytic activities have been cloned and characterized [51,52,53,54,55].

Here we found a significant increase of protease activity (both gelatinolytic and fibrinolytic) in the regenerating tissue after wound clousure from 1 to 14 dpa.

Our results suggested that these metalloproteases could play an essential role in the degradation and remodeling of mesoglea connective tissue during tissue regrowth. These findings are consistent with the results obtained by tissue biometric analysis performed on sections of new formed tentacles 7 days after injury and show that during regeneration the thickness of the mesoglea did not increase, as it did for the epidermis and gastrodermis. However, the consistency of the mesoglea changed visibly and shifted from a loose matrix to a compact one. The change in stiffness was probably due to the ECM remodeling mediated by proteases, secreted by immune cells during wound healing and tissue regeneration phases [56]. The close interplay between the ECM and the innate immune system in cnidarian is mediated by the granular and hyaline amebocytes that use the mesoglea as a territory to move to the wound site, where they infiltrate and aggregate to spread out along the lesion edge that occurs during the inflammation phase in vertebrates [24]. During this process, it is likely that they not only release antimicrobial and cytotoxic material, including melanin, which can destroy pathogen invaders, but they might also produce proteases to facilitated cell migration [57] and secrete new collagen used as a scaffold for tissue reconstruction [33,58].

Our morphological analyses under an optical and electron microscope, and Masson’s trichrome-specific colorimetric assays for ECM, seem to support this hypothesis, showing for the first time that during the tentacle regenerative process, mesoglea shift from a lax network to a lattice organized in bundles of collagen fibrils. Furthermore, immunolocalization assays, using polyclonal antibodies directed against the pro-collagen1α1 and bFGFR antigens, indicate that the visco-elastic behavior of mesoglea is obtained by the neo-synthesis of type I collagen from bFGFR^+^ hyaline amebocytes, acting as fibroblast-like cells. These cells, interacting with the neosynthesized collagen fibrils, appear to be involved in the formation and spatial organization of a compact collagen scaffold.

In conclusion, basing on the obtained evidence, we suggest that in *A. viridis*, the mesoglea ECM provides not only a mechanical support to maintain the shape and integrity of tissues, but also plays a fundamental role in tissue morphogenesis, cell migration and differentiation during tentacle regeneration [59]. Indeed, as already proposed for *N. vectensis*, after tentacle amputation, the change of mesoglea stiffness, obtained through metalloprotease activity, could initially play the role of a barrier against the entry of pathogens into the lesion site. Subsequently, the production of new collagen ECM by fibroblast-like cells, differentiated from amebocytes, could act as a scaffold to guide the correct regrowth of the tentacle. 

## 4. Materials and Methods

### 4.1. Experimental Plan and Reagents

Considering previous studies on the seasonal changes in morphology of *A. viridis*, the specimens have been collected between October and November, when biometric variations are less influenced by thermal stress [37]. Adult organisms were harvested in Termini Imerese, located near Palermo (Italy), maintained at 20 °C under controlled laboratory conditions until experimental use, fed twice a week with mussels and clams and left to acclimate for two weeks before starting the experiments.

Each animal was amputated to the level of 25% of the total tentacles, to be then placed and fed in the tank. After 1, 7 and 14 days after the first amputation, three replicates of animals, respectively, were used for morphological, biometric, biochemical and histological analyses. Not amputated specimens were used as control.

Chemicals were from Sigma-Aldrich (USA).

### 4.2. Tissues Extraction and Estimation of the Proteins Content

For each experimental time, protein extraction was performed separately for body and tentacles cut in the basal zone, near the oral disk using the protocol. Tissues were homogenized in polycarbonate tubes with 10 mL TBS buffer (150 mM NaCl, 10 mM Tris-HCl, pH 7.4) and centrifuged at 12,000 rpm for 30 min at 4 °C (Beckman Allegra 64R Centrifuge, rotor F1202). Supernatant was collected and stored in Eppendorf tubes at −20 °C. The protein content was measured by Bradford method (1976) [60] using bovine serum albumin (BSA) as standard and samples were adjusted at the concentration of 500 µg/mL to perform enzymatic assays.

### 4.3. Alkaline Phosphatase Activity (ALP)

Samples were incubated in an equal volume of 4 mM p-nitrophenyl phosphate liquid in 100 mM ammonium bicarbonate containing 1 mM MgCl_2_ (pH 7.8). The enzymatic kinetic was evaluated [61] at regular intervals of 5 min to 1 h at 405 nm with a microplate reader (RAYTO RT-2100C). One unit (U) of activity was defined as the amount of enzyme required to release 1 micromole of p-nitrophenol produced in 1 min.

### 4.4. Protease Activity

The polyacrylamide electrophoretic run of tentacle and body extracts was performed under non-reducing conditions (7.5%) using gelatin (2 mg/mL) and fibrinogen (0.5 mg/mL) as substrates. Migration took place at 160 V for about 1 h. Gels were washed in TBS Ca^2+^ (150 mM NaCl, 10 mM di Tris-HCl, 10 mM CaCl_2_, pH 8.0) adjusted with 2% Triton X-100. Gels were left to stir overnight in TBS Ca^2+^ (pH 8.0) at room temperature, immersed in 50% methanol, stained in Coomassie Blue (0.1% Coomassie Brillant Blue G250, 40% Methanol, 1% Acetic acid) for an hour and finally decolored (0.1% acetic acid, 0.4% methanol). The reaction was stopped with distilled water and densitometric analysis was carried out using the open-source software Image J (http://rsbweb.nih.gov/ij/download.html) (accessed on 20 May 2021).

### 4.5. Biometry of the Regenerative Area

Animals were placed in a plate and biometric parameters were measured (pedal and oral disc, length of tentacle) using a millimeter gauge, to select specimens of comparable size. Pictures of the part in regeneration were taken using a stereoscopic microscope (Eurotek NB50T, Milano, Italy). The thickness of the different tentacle layers and of the lumen area were calculated in micrometers and then the percentage increase or decrease through mathematical transformation compared to a tentacle of a control animal was also calculated. The biometric evaluation system for tentacles in regeneration was carried out using Image J software. 

### 4.6. Light and Transmission Electron Microscopy (TEM)

#### 4.6.1. Embedding Tissue in Paraffin

The budding area (cutting site with regrowth tissues) of three organisms subjected to the cut of 25% of the total number of tentacles at three times point (1, 7 and 14 dpa) was fixed in Bouin’s solution until complete sinking. Subsequently, samples were dehydrated in an increasing scale of ethanol (30%, 50%, 70%, 90%, 96%, 100%) and paraffin-embedded. Sections (7 μm in thickness) have been cut in series with a rotary microtome (Microm HM 350S, GMBH, Walldorf, Germany) and processed for the Masson’s and Gomori’s and Trichrome stains.

#### 4.6.2. Gomori’s Trichrome

Sections were stained with Carazzi’s emalume (200 mL—10 g KAl(SO_4_), 0.2 g hematoxylin, 0.04 g KIO_3_, 40 mL glycerol) for 10 min at room temperature, washed in distilled water for 15 min, and been left in Gomori’s trichrome solution (100 m—0.6 g chromotropium 2R, 0.3 g fast green FCF, 0.6 g of phosphotungstic acid, 1 mL acetic acid, pH 3.4) and finally washed in acidified water (0.2% acetic acid).

#### 4.6.3. Masson’s Trichrome

Masson’s trichrome assays were performed using a specific colorimetric kit (Trichromica kit, Bio Optica, Milan, Italy) and specimens were treated as suggested by the datasheet, in order to highlight the presence of both collagen and the reticular fibers with aniline blue, while the cell cytoplasm was in red. Images were recorded with a Nikon Digital Sight DS-SM optical Microscope (Nikon, Tokyo, Japan). 

#### 4.6.4. Embedding Tissues in Epoxy Resin 

*A. viridis* tissues, dissected from the area in regeneration, were fixed in 0.1 M cacodylate buffer (pH 7.4) containing 4% glutaraldehyde for 2 h. Specimens were then washed in the same buffer and post-fixed for 1 h with 1% osmium tetroxide in cacodylate buffer (pH 7.4). After standard serial ethanol dehydration, specimens were embedded in an Epon-Araldite 812 mixture. Sections were obtained with a Reichert Ultracut S ultratome (Leica, Wien, Austria). Data were recorded with a DS-5 M-L1 digital camera system (Nikon). Ultrathin sections (0.7 μm in thickness) were placed on copper grids (300 mesh), stained by uranyl acetate and lead citrate and observed with a Jeol 1010 EX transmission electron microscope (Jeol, Tokyo, Japan). 

### 4.7. Immunofluorescence Analyses 

After etching with 3% NaOH in 100% ethanol [62], ultrathin sections were treated pre-incubated for 30 min in BSA blocking solution (2% Bovine Serum Albumin and 0.1% Tween20 in PBS), which was also used to dilute both the primary and secondary antibodies. Subsequently, samples were incubated with anti-COL1α1 (rabbit polyclonal, EMD Millipore, ABT257) and anti-bFGF receptor Flg (rabbit polyclonal, Santa Cruz Biotechnology, sc-121) primary antibodies, diluted 1:200, for 1 h at room temperature. After several washes in PBS (137 mM NaCl, 2.7 mM KCl, 10 mM Na_2_HPO_4_, pH 7.4), specimens were incubated with and goat anti-rabbit Cy3-conjugated secondary antibody (goat, Jackson ImmunoResearch Laboratories, Baltimore Pike, West Grove, PA, USA), diluted 1:200, for 45 min. Cell nuclei were counterstained with DAPI (4,6-diamidino-2-Phenylinedole, 0.1 mg/mL in PBS) for 2 min and slides were mounted with Cityfluor (Cityfluor Ltd., London, UK). Negative control experiments were performed omitting primary antibodies.

### 4.8. Immunogold

Ultrathin sections were obtained as above and collected on gold grids (300 mesh). After etching with NaOH 3% and methanol, specimens were pre-incubated for 30 min in BSA blocking solution and then for 1 h with the anti-bFGF receptor Flg primary antibody, diluted 1:50. After two washings with PBS, the goat anti-rabbit IgG (H + L)-gold conjugate secondary antibody (GE Healthcare, Amersham, UK; particle size, 10 nm), diluted at 1:40, was used for 30 min. In control experiments, the primary antibody was omitted, and sections were incubated with the secondary antibody alone. After washings with PBS, samples were treated for 5 min with 0.5% glutaraldehyde in PBS solution, counterstained with uranyl acetate for 8 min and observed at TEM.

### 4.9. Statistical Analyses 

All the experiments were performed in triplicate and the values used were the mean of the three assays ± SEM. To assess multiple comparisons, a parametric two-way analysis of variance (ANOVA) was performed on the data, with a post hoc Tukey test. Differences between means were considered significant for * *p*  <  0.05, ** *p*  <  0.01 and *** *p*  <  0.001. The analyses were carried out using the GraphPad Prism Version 8.0.0. for Windows (www.graphpad.com) (accessed on 20 May 2021). The mesoglea area and the number of elongated amebocytes were evaluated both in intact and injured tentacles by analyzing five different slides, using the ImageJ software. The software GraphPad Prism 7 (GraphPad Software, La Jolla, CA, USA) was used to perform statistical analyses and differences between treatments were calculated by one-way ANOVA, followed by Fisher’s post-hoc test. Values of *p* < 0.05 were considered statistically significant.

## Figures and Tables

**Table 1:** Two-way ANOVA analysis results of alkaline phosphatase activity (ALP) measured in the tentacles and body extracts of *A. viridis*. Statistically significant effects of the following variables—Time (T) and Injury (I) and interactions (TxI). The analyses were carried out using the GraphPad Prism Version 8.0.0. for Windows (www.graphpad.com (accessed on 1 March 2021)). Differences between means were considered significant for *p* < 0.05.

		Tentacles		Body	
Activity	Variables	*F* Value	*p* Value	*F* Value	*p* Value
ALP	T	1.668	0.229	5.417	0.0089
	I	2.716	0.125	39.83	<0.0001
	T x I	4.996	0.026	6.625	0.003

**Table 2:** Two-way ANOVA analysis results of Proteolytic activities (Fibrinolytic and Gelanolytic) measured in the tentacles and body extracts of *A. viridis*. Statistically significant effects were seen in the following variables—Time (T) and Substrate (S) and interactions (TxS). Bold numbers indicate the significant *p*-value. The analyses were carried out using the GraphPad Prism Version 8.0.0. for Windows (www.graphpad.com (accessed on 1 March 2021)). Differences between means were considered significant for *p* < 0.05.

		Tentacles		Body	
Activity	Variables	*F* Value	*p* Value	*F* Value	*p* Value
PROTEOLYTIC	T	173.5	<0.0001	26.51	<0.0001
	S	4.984	0.00454	1527	<0.0001
	T x S	56.22	<0.0001	102.8	<0.0001

## Data Availability

The authors declare that the data supporting the findings of this study are available within the paper and its supplementary information files. Raw data is available from the corresponding author upon reasonable request.
